# Pennsylvania

**Published:** 1897-02

**Authors:** 


### CONTROL WORK.

*Pennsylvania.*—Philadelphia meat-inspectors during 1896 examined 139,189 head of cattle. Of this number 172 were condemned and destroyed; 142 for tuberculosis, 5 for actinomycosis, and 25 for various other diseases. 53,372 live calves were inspected, of which number 2156 were too young and unfit for slaughter, and their sale prevented.

A herd of 25 cattle at Kennett Square revealed the presence of 15 tuberculous ones, which were condemned by the State Veterinarian; on slaughter all were found to be affected. At Newtown Square, out of a herd of 54, some 42 were condemned and ordered destroyed.

